# Knowledge, Attitudes, and Practices Related to AI in Learning and Research Among Medical Students in Vietnam: Cross-Sectional Study

**DOI:** 10.2196/95867

**Published:** 2026-07-03

**Authors:** Minh Tien Bui, Ha My Le, Thi Khanh Huyen Luong, Hoang Anh Vu, The Anh Tran, Thi Phuong Linh Ngo, Dieu Linh Bui, Thi Han Bui, Duy Cuong Nguyen, Thanh Binh Ngo, Trung Kien Nguyen, Thi Loi Dao

**Affiliations:** 1 Thai Binh University of Medicine and Pharmacy, 373 Ly Bon, Hung Yen, Hưng Yên Province, 410000, Vietnam, 84 2273838545; 2 See Acknowledgments

**Keywords:** artificial intelligence, AI, medical students, knowledge, attitudes, practices

## Abstract

**Background:**

In recent years, artificial intelligence (AI) has ushered in a promising era in medicine, particularly in medical education. However, studies assessing the knowledge, attitudes, and practices related to AI among medical students in Vietnam remain limited.

**Objective:**

This study aimed to evaluate AI knowledge, attitudes, and practices among Vietnamese medical students in learning and research, and to identify factors associated with their AI practices.

**Methods:**

A cross-sectional study was conducted among medical students at Thai Binh University of Medicine and Pharmacy from November to December 2025. Data were collected using an online structured questionnaire covering demographic characteristics and AI knowledge, attitudes, and practices. The main outcome of interest was AI practices in learning and research. Descriptive statistics and multivariable linear regression were used to examine associated factors. Regression coefficients (β), 95% CIs, and *P* values are reported.

**Results:**

A total of 1002 medical students (mean age 21.00, IQR 19.00-23.00 years; n=596, 59.5% female) were included. The median percentage of maximum possible (POMP) score of AI knowledge was 66.67 (IQR 33.33‐83.33), with a high level of familiarity with common tools (n=798, 79.6%). AI attitudes were generally positive (median POMP score 70.00, IQR 53.33‐76.67). AI-related practices were lower (median POMP score 50.00, IQR 46.88-71.88), with AI being used primarily for information retrieval and literature research support. In the multivariable analysis, knowledge POMP score (β=0.12, 95% CI 0.08-0.16) and attitudes POMP score (β=0.42, 95% CI 0.34-0.51) were significantly associated with AI practices POMP score (*P*<.001). Age, gender, major, grade point average classification, and having participated in an AI seminar or training were not associated with AI practices.

**Conclusions:**

Medical students showed favorable knowledge and positive attitudes, but their AI practices remained limited. Integrating AI into medical curricula, including fundamentals, applications, and ethical aspects, is essential to prepare future physicians for AI-driven health care.

## Introduction

Artificial intelligence (AI) is the simulation of human intelligence by machine systems, enabling them to perform tasks that typically require human intelligence [1]. A 2024 global survey on AI practices among students by the Digital Education Council revealed that up to 86% of students used AI in their studies. ChatGPT was the most popular tool (66%), followed by Grammarly and Microsoft Copilot, each accounting for 25% [2].

Globally, the application of AI in medicine and medical education has become widespread and has yielded significant achievements. AI is widely deployed in diagnostic imaging, aiding in the early detection of malignant tumors and supporting physicians in developing personalized treatment regimens [3-5]. Furthermore, AI is gradually affirming its role in health care management and, particularly, in medical education. Recent surveys have consistently reported an increase in the proportion of medical students using AI [6,7]. Most students consider AI a useful tool for optimizing study time and rapidly updating medical knowledge [6,7]. Recent studies have shown that AI is particularly useful in automating repetitive learning tasks, thereby freeing up time for students to focus on cognitive activities and develop complex clinical thinking skills [7,8]. However, studies also indicate that students’ practical knowledge and skills in AI are still very limited. A large survey conducted among 4596 medical, dental, and veterinary students from 192 faculties across 48 countries found that 75.3% of students had little to no AI knowledge [9]. In the same study, 76.3% reported that their curricula did not include any courses or lectures on this topic [9]. In Vietnam, AI has enormous potential to improve the quality of medical education. However, its current application is limited due to shortcomings in infrastructure, policies, faculty readiness, and foundational AI knowledge [10]. A survey in Southern Vietnam reported that 92.2% of students lacked understanding of AI in health care [11]. On the other hand, Vietnamese medical students have also expressed concern about a potential “replacement crisis.” This refers to the fear that the professional knowledge and skills they are developing could become obsolete or be replaced by AI [12]. Medical students have expressed concerns that AI could diminish professional competence, critical thinking, and clinical intuition. These are key foundations of medical practice and may be weakened if students rely too heavily on algorithms [13].

Despite numerous efforts and implementation activities, no comprehensive study has assessed the current state of AI knowledge, attitudes, and practices among Vietnamese medical students to date. As AI is increasingly becoming an essential requirement in medical education, assessing this reality is crucial. Such assessment can provide universities with evidence to develop suitable training programs, strengthen students’ technological capacity, and guide the effective and safe use of AI in learning and research. Therefore, we conducted this study to evaluate the AI knowledge, attitudes, and practices of Vietnamese medical students in learning and research and to identify factors associated with their AI practices.

## Methods

### Study Design and Participants

This cross-sectional descriptive study was conducted at Thai Binh University of Medicine and Pharmacy, Vietnam, during the 2025 to 2026 academic year. Data were collected from November 22 to December 31, 2025.

Participants included medical students from the first year to the sixth year enrolled in 3 programs: general medicine, traditional medicine, and preventive medicine. Thai Binh University of Medicine and Pharmacy is a key medical training institution in Northern Vietnam and in the country, offering various programs in the health sciences. Among these, students enrolled in general medicine, traditional medicine, and preventive medicine account for nearly 70% of all undergraduate students. Furthermore, these 3 specialties share similar training objectives and curricular structures. In addition, they provide foundational knowledge in basic medical sciences, clinical medicine, and public health. Therefore, selecting participants from these 3 programs helped ensure homogeneity of the study population and enhanced the comparability of the results. This selection was feasible given the available time and resources for study implementation.

Using a conservative expected proportion of good knowledge of 50%, a 95% CI, and a 5% absolute precision, the minimum required sample size was 385. The final sample of 1002 students exceeded this requirement, improving statistical power and precision.

### Data Collection Tool

Data were collected using a 36-item self-administered online questionnaire developed based on the knowledge, attitude, and practice (KAP) framework and previous studies [11,14-16] (Multimedia Appendix 1).

The questionnaire consisted of 4 sections. The first section collected participants’ sociodemographic characteristics, previous knowledge, and AI use, including age, gender, academic profile (major and grade point average [GPA] classification), and previous participation in AI-related seminars or training. The second section assessed AI knowledge through 6 questions. Each “yes” answer counts for 1 point, with a maximum score of 6 points (Multimedia Appendix 1). The third section evaluated AI attitudes using 15 questions on a 5-point Likert scale ranging from strongly disagree to strongly agree, with total scores ranging from 15 to 75 (Multimedia Appendix 1). The fourth section examined AI practices through 8 questions, also based on a 5-point Likert scale ranging from never to always, with total scores ranging from 8 to 40 (Multimedia Appendix 1).

The questionnaire was pilot-tested on 40 students. Internal consistency was acceptable with Cronbach α values of 0.69 for knowledge, 0.90 for attitudes, and 0.83 for practices. No changes were made to the questionnaire after pilot testing, as no major issues were identified regarding item clarity, comprehension, or completion. In addition, the internal consistency of the 3 domains was acceptable. Data from the pilot test were excluded from the final analysis.

The Cronbach α in this study was 0.73 for knowledge, 0.95 for attitudes, and 0.92 for practices.

### Procedures

Sampling was conducted using the convenience sampling method. The questionnaire was designed using Google Forms with an estimated completion time of 5 to 10 minutes. The survey link and QR code were distributed via student groups on Zalo (VNG Corporation) and Facebook (Meta Platforms). All students from 3 programs were invited to participate. In the introduction to the questionnaire, we explained the objectives of the study and the estimated time required to complete the questionnaire. Only students who agreed to participate in the study were allowed to answer the research questionnaire. Participation was entirely voluntary, and students could exit the survey at any time without penalty. To reduce social desirability bias inherent to self-report surveys, responses were anonymous. No academic identifiers were collected, and students were explicitly informed that their answers would not affect their academic evaluation. All responses were automatically recorded and stored for data cleaning and analysis. Only participants who completed all survey questions were included in the final analysis.

### Study Variables

The main outcome of interest in this analysis was AI practices, measured using the percentage of maximum possible (POMP) score, calculated using the formula: (observed score–minimum possible score)/(maximum possible score–minimum possible score)×100 [17]. AI knowledge and AI attitudes were also expressed as POMP scores. All POMP scores ranged from 0 to 100, with higher scores indicating better AI practices, better knowledge, or more positive attitudes toward AI.

Potential confounders were selected a priori based on theoretical relevance and included age (continuous, in years), gender (male, female, or other), major (general medicine, traditional medicine, or preventive medicine), GPA classification (excellent, very good, good, average, or poor), and previous participation in AI-related seminars or training (yes or no).

### Statistical Analysis

All analyses were performed using RStudio (version 4.6.0). Categorical variables were summarized as frequencies and percentages. Continuous variables were summarized as means and SDs or medians and IQRs, as appropriate.

Our main outcome of interest was AI practices as a continuous variable (POMP score). To examine the association between knowledge and practices, we fitted a standard multivariable linear regression model. The AI practices were used as the dependent variable. The AI knowledge POMP score was the main explanatory variable. All covariates were entered into the model concurrently. The model was adjusted for AI attitude POMP score, age, gender, major, GPA classification, and previous participation in AI seminars or training. Regression coefficients (β), 95% CIs, and *P* values were reported. The *R*^2^ and adjusted *R*^2^ were also reported to describe the proportion of variance explained by the final model.

As diagnostic plots suggested mild departures from normality and homoscedasticity of residuals, robust SEs were used in the final model estimates. Specifically, heteroskedasticity-consistent robust SEs (HC3) were applied to improve the validity of statistical inference.

Model assumptions were assessed using standard diagnostic plots, including residuals versus fitted values, normal Q-Q, scale-location, and residuals versus leverage plots (Multimedia Appendix 2). Potentially influential observations were identified using Cook distance. As a sensitivity analysis, observations with a Cook distance greater than 4/n were excluded, and the multivariable model was refitted to assess the robustness of the main findings. The primary analysis was based on the full sample, while the reduced-sample model was used only for sensitivity assessment.

All statistical tests were 2-sided, and a *P* value of <.05 was considered statistically significant.

### Ethical Considerations

The protocol was approved by the Thai Binh University of Medicine and Pharmacy Institutional Review Board (reference number: 2240; approval date: November 21, 2025). Before answering the questionnaire, participants were clearly informed about the research objectives, the number of questions, the research unit, and how the collected data would be stored. Participants had the right to decline participation. They were required to provide voluntary informed consent before completing the questionnaire. Participants were informed that no identifiable personal information would be collected and that their data would be used solely for research purposes. No compensation was provided.

## Results

### Characteristics of the Study Population

A total of 1002 students were included in the analysis. The median age was 21.00 years. Female students accounted for the majority of the sample, while most participants were enrolled in general medicine. Regarding academic performance, more than half of the students were classified as having a good GPA, followed by those with an average or poor GPA (Table 1).

**Table 1.** Demographic and academic characteristics of participants (N=1002).

Characteristic	Values
Age (years), median (IQR)	21.00 (19.00‐23.00)
Gender, n (%)	
Male	400 (39.9)
Female	596 (59.5)
Other	6 (0.6)
Major, n (%)	
General medicine	783 (78.1)
Traditional medicine	97 (9.7)
Preventive medicine	122 (12.2)
GPA^a^ classification, n (%)	
Excellent	28 (2.8)
Very good	166 (16.6)
Good	581 (58.0)
Average or poor	227 (22.6)
Participated in an AI^b^ seminar or training, n (%)	
Yes	292 (29.1)

a GPA: grade point average.

b AI: artificial intelligence.

### AI Knowledge of Medical Students

The knowledge level was moderate, with a median knowledge score of 66.66. Familiarity was highest for AI tools used in education, with 79.6% (798/1002) of participants reporting that they were familiar with tools such as ChatGPT, Gemini, or Bing. More than half of the students were aware of the applications of AI in health care and had a good understanding of the concept of AI in education (Table 2).

**Table 2.** Artificial intelligence (AI) knowledge of medical students (N=1002)^a^.

Characteristic	Responses, n (%)
Do you have a good understanding of the basics of AI?	
No	369 (36.8)
Yes	633 (63.2)
Do you know what deep learning and machine learning are?	
No	747 (74.6)
Yes	255 (25.4)
Are you aware of any applications of AI in health care?	
No	432 (43.1)
Yes	570 (56.9)
Do you understand the barriers to applying AI in learning and research?	
No	294 (29.3)
Yes	708 (70.7)
Do you have a clear understanding of the concept of AI in education?	
No	457 (45.6)
Yes	545 (54.4)
Are you familiar with different AI tools used for educational purposes (ChatGPT, Gemini, Bing, etc)?	
No	204 (20.4)
Yes	798 (79.6)

a Median knowledge score was 66.66 (IQR 33.33-83.33).

### AI Attitudes of Medical Students

**Figure 1.**
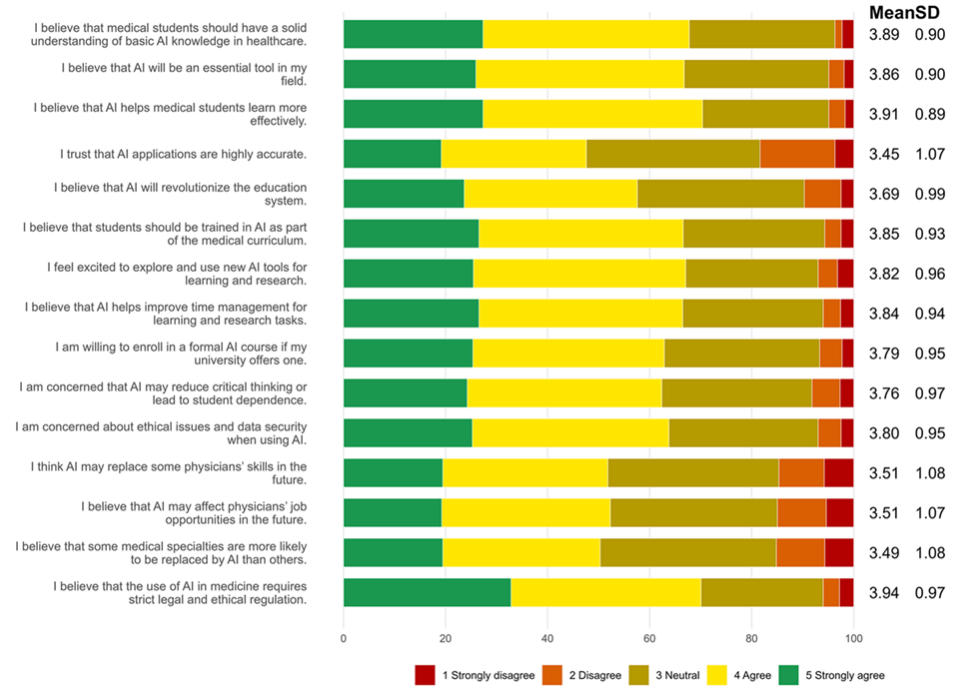
Attitude of medical students regarding artificial intelligence (AI) use in training and research.

Overall, students showed generally positive AI attitudes, with mean item scores ranging from 3.45 to 3.94 out of 5. Agreement was strongest for the need for legal and ethical regulation of AI and for the importance of AI knowledge in medical education, while lower scores were observed for trust in AI accuracy and concerns about AI replacing physician roles (Figure 1). The attitude POMP score was 70.00 (IQR 53.33‐76.67).

### AI Practices of Medical Students

**Figure 2.**
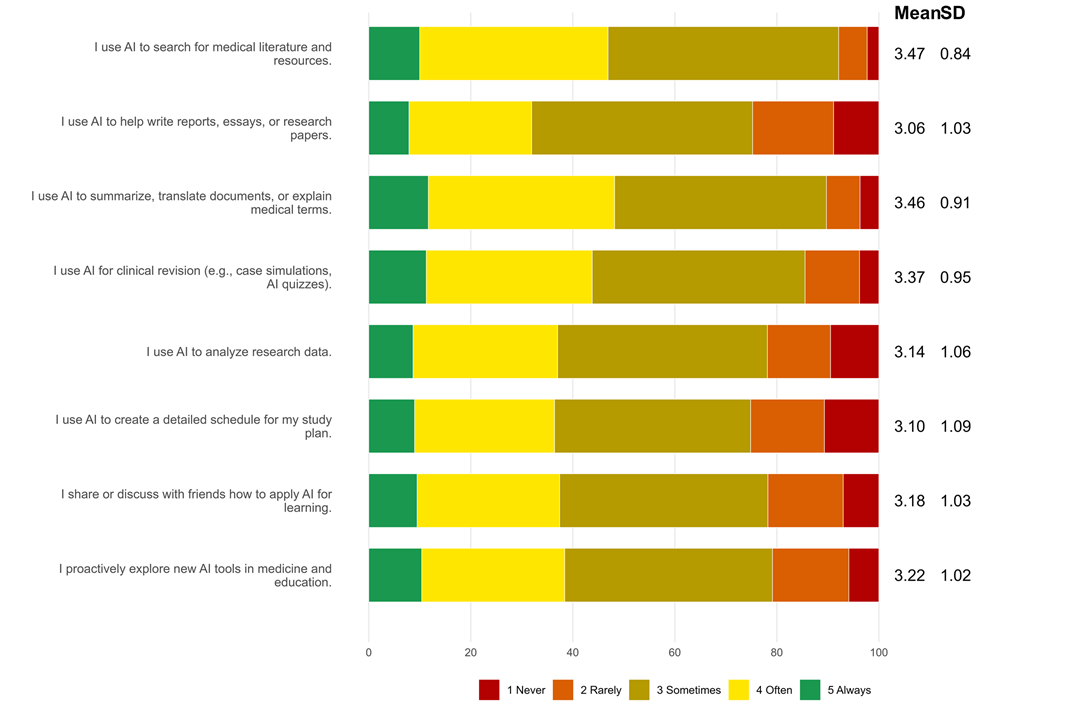
Practice of medical students regarding artificial intelligence (AI) use in training and research.

Students reported moderate AI practices, with mean item scores ranging from 3.06 to 3.47 out of 5. The most common uses were searching for medical literature and resources and summarizing or translating documents, while less frequent uses included writing reports or research papers, creating detailed study schedules, and analyzing research data. These findings suggest that students mainly used AI for supportive and information-oriented tasks rather than for more advanced academic or analytic activities (Figure 2). The AI practices POMP score was 50.00 (IQR 46.88‐71.88).

### Factors Associated With AI Practices of Medical Students

In the multivariable linear regression, both knowledge score and attitude score were positively associated with AI practice score. In the main analysis, each 1-point increase in the knowledge POMP score was associated with a 0.12-point increase in the practice score, while each 1-point increase in the attitude POMP score was associated with a 0.42-point increase in the AI practice score. These associations remained significant in the sensitivity analysis, with slightly larger effect estimates for both knowledge and attitude (Table 3).

Most of the other covariates were not significantly associated with AI practice scores in either model. Age was not associated with practice scores in the main analysis but became positively associated in the sensitivity analysis (Table 3).

**Table 3.** Associated factors with artificial intelligence (AI) practices of medical students.

Variables		Main analysis^a^, β (95% CI)	P value	Sensitivity analysis^b^, β (95% CI)	P value
Knowledge score (POMP^c^)		0.12 (0.08 to 0.16)	<.001	0.13 (0.10 to 0.16)	<.001
Attitude score (POMP)		0.42 (0.34 to 0.51)	<.001	0.44 (0.38 to 0.50)	<.001
Age (y)		0.05 (–0.44 to 0.54)	.84	0.42 (0.05 to 0.79)	.03
Gender (reference: male)					
	Female	–0.97 (–3.26 to 1.32)	.41	–1.75 (–3.73 to 0.24)	.08
	Other	–11.08 (–33.11 to 10.96)	.32	–15.02 (–20.95 to –9.10)	<.001
Major (reference: general medicine)					
	Traditional medicine	–3.14 (–7.44 to 1.16)	.15	–3.17 (–6.83 to 0.48)	.09
	Preventive medicine	–2.50 (–6.47 to 1.47)	.22	–1.50 (–4.80 to 1.80)	.37
GPA^d^ classification (reference: excellent)					
	Very good	2.34 (–4.25 to 8.92)	.49	3.24 (–1.57 to 8.04)	.19
	Good	1.33 (–4.91 to 7.56)	.68	2.09 (–2.38 to 6.56)	.36
	Average or poor	1.55 (–5.01 to 8.11)	.64	2.50 (–2.29 to 7.28)	.31
Participated in AI seminar/training (reference: no)					
	Yes	1.06 (–1.48 to 3.59)	.41	0.48 (–1.78 to 2.74)	.68

a Multiple *R*2=0.22; adjusted *R*2=0.21.

b Multiple *R*2=0.29; adjusted *R*2=0.28.

c POMP: percentage of maximum possible.

d GPA: grade point average.

## Discussion

### Principal Findings

This study provides an overview of AI knowledge, attitudes, and practices in learning and research among Vietnamese students and identifies factors associated with their AI practices. Overall, students showed relatively favorable AI knowledge and attitudes. However, their actual AI practices appeared more limited. This suggests a gap between awareness, perception, and practical application. The findings also indicate that students with better knowledge and more positive attitudes were more likely to report greater AI practices. In contrast, demographic and academic characteristics appeared to play a less important role. These results highlight the importance of improving both AI literacy and confidence in applying AI tools in medical education.

### Comparison With Prior Work and Interpretation of Findings

In the context of the digital revolution reshaping every aspect of life, global medical education is no exception, and it has made significant strides in the application of AI. Medical students worldwide are increasingly proactively integrating AI into their learning processes, viewing it as a powerful tool to optimize knowledge acquisition.

In this study, most students reported familiarity with AI tools used in education, such as ChatGPT and Gemini, and many indicated an understanding of basic AI concepts. However, more advanced knowledge remained limited, with only approximately one-quarter of participants reporting an understanding of concepts such as machine learning and deep learning. This pattern suggests that students are more likely to engage with AI as end users in daily learning activities rather than to possess a deeper conceptual understanding of the underlying technologies. These findings are consistent with previous international studies. A study of 762 medical students in Sudan found that 15.7% lacked knowledge of basic AI concepts, 67.3% had no knowledge of deep learning and machine learning, and 56.7% were unaware of any applications of AI in the health care field [16]. Previous studies conducted among medical students in Vietnam also reported similar results [11,13]. In one study, 91% of students had never received formal training in AI use [13]. Another study found that 92.2% had no understanding of AI in medicine, and 89.1% had never attended any AI-related seminars or specialized courses [11]. One possible explanation is that AI has not yet been systematically integrated into the medical curriculum in many training institutions. Consequently, students often acquire AI knowledge through informal sources such as social media, online platforms, or discussions with friends, rather than through structured educational programs or experts in the field. While this informal exposure may increase familiarity with widely accessible AI tools, it may not be sufficient to develop a comprehensive understanding of core AI principles or their clinical applications. These findings highlight the need to incorporate formal AI training into medical education. Such training should focus on fundamental concepts, including machine learning, data analytics, and AI application in clinical medical practice.

In our study, students generally demonstrated positive AI attitudes, indicating a high level of AI acceptance among medical students. Most students agreed that AI knowledge is crucial in medical education and stressed the need for legal and ethical regulations governing the use of AI in health care. These findings are consistent with studies from various countries [7,8,18-21]. Previous research showed that medical students recognize the potential of AI to support clinical decision-making, improve learning outcomes, and enhance patient care quality [7,8,18-21].

However, students also expressed concerns about the reliability of AI-generated information and the possibility that AI could replace some traditional roles performed by physicians. This ambivalence has also been reported in previous studies, with students recognizing the benefits of AI while remaining concerned about its potential risks [13,19,22,23]. A study in India found that 37.6% of students believed that AI could replace medical professionals. A proportion of 69.2% participants were concerned that the human aspect and empathy in medicine would be diminished [19]. Ethical, security, and reliability issues were also major hurdles. A study in China indicated that 78.3% of medical students were concerned that AI would not guarantee user privacy. In addition, 84.8% expressed concerns about the accuracy of AI-generated results [22]. Another study among Arab medical students reported that 36.1% believed that AI would reduce the number of radiologists in the future [23]. In Vietnam, a large proportion of medical students feared that overreliance on AI could reduce critical thinking skills or clinical intuition [13].

These concerns may reflect the limited formal training on AI. Students may need clearer guidance on its capabilities, limitations, ethical implications, and appropriate clinical use. Without sufficient education, students may perceive AI as both highly promising and potentially threatening to professional identity and patient-centered care. Overall, the positive attitudes observed in this study suggest that students are receptive to integrating AI into medical education. However, implementation should be supported by appropriate ethical frameworks, data governance policies, and professional standards. Despite having favorable knowledge and positive attitudes, students demonstrated limited AI practices. This suggests a gap between AI awareness, acceptance, and actual practical application in academic settings. The most common applications of AI tools included medical literature retrieval, document translation, and academic document summarization. Conversely, more advanced applications such as research report writing, study planning, or research data analysis were reported less frequently. Similar findings have been reported in other studies where AI tools are commonly used for text summarization, assignment drafting, or language editing assistance [8,24-27]. In our study, searching for medical literature and summarizing or translating documents were the most frequently reported practices. This aligns with the findings from a survey of 550 medical students in Palestine conducted by Yousef M et al [8]. However, compared with the Palestinian cohort, AI use for research-related tasks in our sample appeared relatively lower, particularly in writing research papers, analyzing research data, and creating a detailed study plan. This difference may reflect variations in research exposure, digital literacy, or institutional integration of AI training across educational settings. Several factors may explain this pattern. Limited knowledge of advanced AI capabilities may restrict students’ ability to apply AI to more complex tasks. Furthermore, students may lack guidance on how to effectively integrate AI into their research processes. In addition, limited access to structured training or mentorship may reduce students’ confidence in applying AI to academically demanding tasks. On the other hand, concerns about academic integrity and the potential for misuse of AI-generated content may also deter students from using AI to write scientific papers.

These findings underscore the importance of providing training on responsible and effective AI use in academic and research contexts. Training should emphasize practical applications such as evidence synthesis, data analysis, scientific writing, and critical appraisal of AI-generated outputs. Such training may help bridge the gap between positive perceptions of AI and its meaningful use in academic and research practice. It may also help maintain academic integrity and professional standards.

Our research findings demonstrate that both knowledge and attitudes are significantly associated with AI practices. These findings are consistent with the KAP theoretical framework, which proposes that knowledge influences attitudes, and both knowledge and attitudes contribute to shaping practical behaviors. In this context, students with a better understanding of AI and a more positive perception of its use are more likely to integrate AI tools into their learning and research activities. Our results are consistent with previous studies worldwide and further support the important role of knowledge and attitudes in AI practices [28,29]. Indeed, a study by Ghanem et al [28] conducted among 423 medical students observed a positive correlation between the level of knowledge and satisfactory practice levels. The study by Alabbad et al [29] also reported statistically significant positive correlations between knowledge and practices, and between attitudes and practices.

From an educational perspective, this relationship may be particularly relevant in medical training, where students are continuously exposed to large volumes of information, evidence-based decision-making, and research-related tasks. Students with higher AI-related knowledge may better appreciate the potential of AI in supporting literature retrieval, summarizing scientific information, generating study materials, or assisting with data analysis. This improved understanding may foster more positive AI perceptions and support more frequent and diverse use. Conversely, students with limited knowledge may perceive AI as complex, unreliable, or ethically concerning, which could hinder active adoption despite the availability of these tools. Importantly, these findings also carry practical implications for medical education. Improving AI practices may not depend solely on providing access to AI tools but rather on developing foundational AI literacy and fostering constructive attitudes toward responsible AI use. Structured educational interventions may help bridge the gap between awareness and effective implementation. These interventions could include integrating AI concepts into undergraduate curricula, offering hands-on workshops, and promoting ethical discussions on AI applications in medicine. Such approaches may help students use AI more confidently, critically, and responsibly in their future professional practice.

Interestingly, most demographic and academic characteristics, including gender, major, GPA, and participation in AI-related workshops, were not significantly associated with AI practices. Similar results were reported in a study of 410 medical students in Greece, which showed that the use of AI in medical education or medical practice was not significantly associated with student characteristics [30]. This suggests that individual cognitive and perceptual factors may play a more significant role than demographic characteristics in determining the extent to which medical students adopt AI tools. However, in the sensitivity analysis, age showed a small but statistically significant association with practice scores, suggesting that older students may be slightly more inclined to use AI in learning activities. Although the effect size was relatively small, this finding may still have educational relevance. This could reflect greater academic experience, higher self-directed learning capacity, or increased exposure to research activities during the later years of medical training.

To our knowledge, this is the first comprehensive study surveying the KAP framework regarding AI use on a relatively large sample size of medical students in Northern Vietnam. Therefore, the results provide relatively precise estimates. Furthermore, the inclusion of students from all years of study and from 3 medical programs ensures relative representativeness and comparability within the research context. In addition, the questionnaire was developed based on the KAP framework and previous studies, with good internal consistency, supporting the reliability of the measurements. Moreover, the use of standardized POMP scores facilitated the interpretation and comparison of results. The application of multivariable regression adjusted for potential confounding factors, along with robust SEs and sensitivity analysis, further strengthened the validity of the findings.

### Limitations

Our study has some limitations. First, the use of self-reported data may have introduced bias due to the subjective recall and self-assessment of the study participants. Second, as the study was conducted at a single medical university, the generalizability of the findings to other contexts may be limited. Third, the online, voluntary survey method relied on convenience sampling, which may have introduced selection bias and affected the external validity of the study. Students with greater interest in AI, higher digital literacy, or stronger academic engagement may have been more likely to participate. This could potentially lead to an overestimation of AI knowledge, attitudes, or practices among the study population. Larger studies across multiple medical schools are needed to confirm these findings and inform the integration of AI education into medical training in context-appropriate ways.

### Conclusions

The findings of this study highlight the need for medical education to move beyond students’ informal exposure to AI tools and toward a more structured approach to AI literacy. As AI becomes increasingly embedded in health care, research, and academic work, future physicians will require not only familiarity with available tools but also the knowledge and judgment to use them critically, ethically, and effectively. Strengthening these competencies during medical training may help ensure that AI supports, rather than undermines, the quality, integrity, and accountability of future clinical and research practice.

Medical schools should therefore consider integrating AI-related learning into undergraduate curricula through a combination of foundational concepts; practical applications; and discussions of ethical, legal, and professional responsibilities. Such training could include AI principles, the appropriate use of AI tools for learning and research, the critical appraisal of AI-generated outputs, and responsible AI use in health care contexts. More broadly, developing clear educational guidance and strengthening faculty capacity in this area will be important for preparing a medical workforce that can engage with AI thoughtfully and adapt to its expanding role in medicine.

## Supplementary material

10.2196/95867 Multimedia Appendix 1 Data collection tool.

10.2196/95867 Multimedia Appendix 2 Diagnostic plots for the multivariable linear regression model assessing the association between knowledge score and practice score for artificial intelligence use in learning and research.

10.2196/95867 Checklist 1 STROBE checklist.

## Abbreviations

AI: artificial intelligence
GPA: grade point average
KAP: knowledge, attitude, and practice
POMP: percentage of maximum possible
