# Supplementary material for: Knowledge, Attitudes, and Practices Related to AI in Learning and Research Among Medical Students in Vietnam: Cross-Sectional Study
Source: JMIR Form Res. 2026 Jul 3;10:e95867. doi: 10.2196/95867 (PMC13330737; doi:10.2196/95867)
Supplement: Multimedia Appendix 1 [file formative-v10-e95867-s001.docx]

**KNOWLEDGE, ATTITUDES, AND PRACTICES ON ARTIFICIAL INTELLIGENCE IN LEARNING AND RESEARCH AMONG MEDICAL STUDENTS AT THAI BINH UNIVERSITY OF MEDICINE AND PHARMACY**

Before continuing, please carefully read the following information:

**Purpose of the study:** This study aims to assess the knowledge, attitudes, and practices of AI in the learning and research of medical students at Thai Binh University of Medicine and Pharmacy.

**Voluntary participation:** Participation in this study is entirely voluntary. You have the right to refuse to answer any questions or withdraw from the study at any time.

**Confidentiality:** No identifiable personal information will be collected. All responses will be anonymous and kept strictly confidential. Data collected will only be used for research purposes and will be stored securely.

Duration: Completing this questionnaire will take approximately 5-10 minutes.

By completing this questionnaire, you agree to participate in this study.

☐ Yes

☐ No

| **General information section:** | | | | | | | | | | | |
| --- | --- | --- | --- | --- | --- | --- | --- | --- | --- | --- | --- |
| 1 | | Gender | | ☐ Male ☐ Femal ☐ Other | | | | | | | |
| 2 | | Age | | ………….. | | | | | | | |
| 3 | | Major | | ☐ General medicine ☐ Traditional medicine ☐ Preventive medicine | | | | | | | |
| 4 | | Year | | ☐ Y1  ☐ Y2  ☐ Y3  ☐ Y4  ☐ Y5  ☐ Y6 | | | | | | | |
| 6 | | Grade point average classification from previous semesters to the present | | ☐ Excellent  ☐ Very good ☐ Good ☐ Average  ☐ Poor | | | | | | | |
| 7 | | Previous participation in AI-related seminars or training? | | ☐ Yes ☐ No | | | | | | | |
| Section assesses knowledge of AI | | | | | | | | | | |  |
| K1 | | | Do you have a good understanding of the basics of AI? | | | | ☐ No  ☐ Yes | | | |  |
| K2 | | | Do you know what deep learning and machine learning are? | | | | ☐ No  ☐ Yes | | | |  |
| K3 | | | Are you aware of any applications of AI in healthcare? | | | | ☐ No  ☐ Yes | | | |  |
| K4 | | | Do you understand the barriers to applying AI in learning and research? | | | | ☐ No  ☐ Yes | | | |  |
| K5 | | | Do you have a clear understanding of the concept of AI in education? | | | | ☐ No  ☐ Yes | | | |  |
| K6 | | | Are you familiar with different AI tools used for educational purposes (ChatGPT, Gemini, Bing, etc.)? | | | | ☐ No  ☐ Yes | | | |  |
| Section assesses attitude of AI  **Please indicate your level of agreement with the following statements: 1 – Strongly disagree, 2 – Disagree, 3 – Neutral, 4 – Agree, 5 – Strongly agree.** | | | | | | | | | | |  |
| Questions | | | | | **I strongly disagree with this.** | **I disagree with this.** | | **I neutral with this.** | **I agree with this.** | **I strongly agree with this.** |  |
| A1 | I believe that medical students should have a solid understanding of basic AI knowledge in healthcare. | | | |  |  | |  |  |  |  |
| A2 | I believe that AI will be an esential tool in my field. | | | |  |  | |  |  |  |  |
| A3 | I believe that AI helps medical students learn more effectively | | | |  |  | |  |  |  |  |
| A4 | I trust that AI applications are highly accurate. | | | |  |  | |  |  |  |  |
| A5 | I believe that AI will revolutionize the education system. | | | |  |  | |  |  |  |  |
| A6 | I believe that students should be trained in AI as part of the medical curriculum. | | | |  |  | |  |  |  |  |
| A7 | I feel excited to explore and use new AI tools for learning and research. | | | |  |  | |  |  |  |  |
| A8 | I believe that AI helps improve time management for learning and research tasks. | | | |  |  | |  |  |  |  |
| A9 | I am willing to enroll a forrmat AI course if my university offers one. | | | |  |  | |  |  |  |  |
| A10 | I am concerned about ethical issues and data security when using AI | | | |  |  | |  |  |  |  |
| A11 | I am concerned that AI may reduce critical thinking or lead to student dependence. | | | |  |  | |  |  |  |  |
| A12 | I think AI may replace some physicians’ skills in the future. | | | |  |  | |  |  |  |  |
| A13 | I believe that AI may affect physicians’ job opportunities in the future. | | | |  |  | |  |  |  |  |
| A14 | I believe that some medical specialties are more likely to be replaced by AI than others. | | | |  |  | |  |  |  |  |
| A15 | I believe that the use of AI in medicine requires strict legal and ethical regulation. | | | |  |  | |  |  |  |  |
| Section assesses practice of AI  **Please indicate the frequency and extent to which you use it: 1 – Never, 2 – Rarely, 3 – sometimes, 4 – often, 5 – Always.** | | | | | | | | | | |  |
| Câu hỏi | | | | | 1 – Never | 2 – Rarely | | 3 – Sometimes | 4 – Often | 5 - Always |  |
| P1 | I use AI to search for medical literature and resources. | | | |  |  | |  |  |  |  |
| P2 | I use AI to help write reports, essays, or research papers. | | | |  |  | |  |  |  |  |
| P3 | I use AI to summarize, translate documents, or explain medical terms. | | | |  |  | |  |  |  |  |
| P4 | I use AI for clinical revision (case simulation, AI quizzes). | | | |  |  | |  |  |  |  |
| P5 | I use AI to analyze research data. | | | |  |  | |  |  |  |  |
| P6 | I use AI to create a detailed schedule for my study plan. | | | |  |  | |  |  |  |  |
| P7 | I share or discuss with friends how to apply AI for learning. | | | |  |  | |  |  |  |  |
| P8 | I proactively explore new AI tools in medicine and education. | | | |  |  | |  |  |  |  |
